# Large-Area Growth of Uniform Single-Layer MoS_2_ Thin Films by Chemical Vapor Deposition

**DOI:** 10.1186/s11671-015-1094-x

**Published:** 2015-10-06

**Authors:** Seung Hyun Baek, Yura Choi, Woong Choi

**Affiliations:** School of Advanced Materials Engineering, Kookmin University, Seoul, 02707 South Korea

**Keywords:** MoS_2_, Single layer, Thin films, Chemical vapor deposition, 81.05.Hd, 81.10.Bk, 81.15.Gh

## Abstract

We report the largest-size thin films of uniform single-layer MoS_2_ on sapphire substrates grown by chemical vapor deposition based on the reaction of gaseous MoO_3_ and S evaporated from solid sources. The as-grown thin films of single-layer MoS_2_ were continuous and uniform in thickness for more than 4 cm without the existence of triangular-shaped MoS_2_ clusters. Compared to mechanically exfoliated crystals, the as-grown single-layer MoS_2_ thin films possessed consistent chemical valence states and crystal structure along with strong photoluminescence emission and optical absorbance at high energy. These results demonstrate that it is possible to scale up the growth of uniform single-layer MoS_2_ thin films, providing potentially important implications on realizing high-performance MoS_2_ devices.

**Electronic supplementary material:**

The online version of this article (doi:10.1186/s11671-015-1094-x) contains supplementary material, which is available to authorized users.

## Background

Two-dimensional transition metal dichalcogenides (TMDs) have received great attention because of their interesting electronic, optical, and chemical properties. Among various TMDs, molybdenum disulfide (MoS_2_) has been most extensively investigated for the applications of thin-film transistors (TFTs), photodetectors, and energy storage [1–3]. TFTs based on single- or multilayer MoS_2_ exhibit intriguing transistor performance including high on/off current ratio (~10^7^), high mobility at room temperature (~100 cm^2^ V^−1^ s^−1^), and low subthreshold swing (~70 mV decade^−1^) [4, 5]. Moreover, photodetectors based on single- or multilayer MoS_2_ show high photoresponsivity (300–800 A W^−1^) exceeding that of silicon-based ones [6, 7]. However, the aforementioned examples have been demonstrated using mechanically exfoliated MoS_2_ flakes, which are typically micrometer-scale in size. Hence, the growth of large-area MoS_2_ is one of the critical challenges to realize its promising potential.

So far, a variety of synthesis approaches have been reported to grow large-area MoS_2_ including liquid exfoliation (sonication in solvents) [8], two-step chemical vapor deposition (CVD, sulfurization or decomposition of pre-deposited Mo-based thin films) [9–11], one-step CVD (reaction of gaseous Mo and S precursors) [12–14], and physical vapor deposition (sputtering and pulsed laser deposition) [15, 16]. Special emphasis has been put on one-step CVD as it shows greater potential for growing uniform large-grain thin films of single-layer MoS_2_. The most common one-step CVD is based on the reaction of gaseous MoO_3_ and S evaporated from solid sources due to the simplicity of processing and the easy availability of solid sources [12]. When the optimized CVD process conditions with MoO_3_ and S precursors are combined with the use of mica or substrate treatment, the formation of single-layer MoS_2_ thin films can be obtained up to about a centimeter [17–19]. However, CVD processes based on MoO_3_ and S powders typically result in triangular-shaped discontinuous clusters of either single-layer MoS_2_ or mixtures of single- and few-layer MoS_2_ [12]. Therefore, more work is needed to establish a CVD process that can reproducibly provide continuous large-area thin films of uniform single-layer MoS_2_.

Here, we investigate CVD methods based on MoO_3_ and S powders to grow continuous thin films of single-layer MoS_2_ for more than 4 cm on sapphire substrates. Our MoS_2_ thin films are the largest in size grown by CVD methods based on MoO_3_ and S powders. The large-area deposition, thickness uniformity, and crystallinity of single-layer MoS_2_ thin films are confirmed by scanning electron microscopy (SEM), atomic force microscopy (AFM), x-ray photoelectron spectroscopy (XPS), Raman spectroscopy, photoluminescence (PL) spectroscopy, ultraviolet (UV)-visible spectroscopy, and transmission electron microscopy (TEM).

## Methods

MoS_2_ films were deposited on (0001)-oriented sapphire substrates in a two-zone tube furnace. MoO_3_ (99.98 %, Sigma-Aldrich) and S (99.98 %, Sigma-Aldrich) powders in two separate Al_2_O_3_ boats were used as precursors. MoO_3_ powder (15 mg) was placed upstream at zone 1 (700 °C), and S powder (1 g) was placed at the upstream entry of the furnace. The substrates were placed downstream at zone 2 (600 °C). MoO_3_ powder was heated up to 700 °C at a rate of 15 °C min^−1^, and the substrates were heated up to 600 °C at 38 °C min^−1^. After 30-min deposition, the furnace was slowly cooled down to room temperature. Ar flow of 100 sccm and a pressure of ~0.5 Torr were maintained during deposition.

The surface morphology of deposited thin films was observed by SEM (JEOL JSM-7610F) and AFM (Park Systems XE-100). Elemental composition was analyzed using XPS (PHI X-tool). The thickness and uniformity of deposited thin films were measured by Raman and PL spectra (Horiba LabRAM Aramis) using a laser of 532 nm in wavelength. Optical absorbance was measured by UV-visible spectroscopy (PerkinElmer Lambda 35). Crystal structure was analyzed by TEM (FEI Titan 80–300) at 300 kV.

## Results and Discussion

**Fig. 1:**
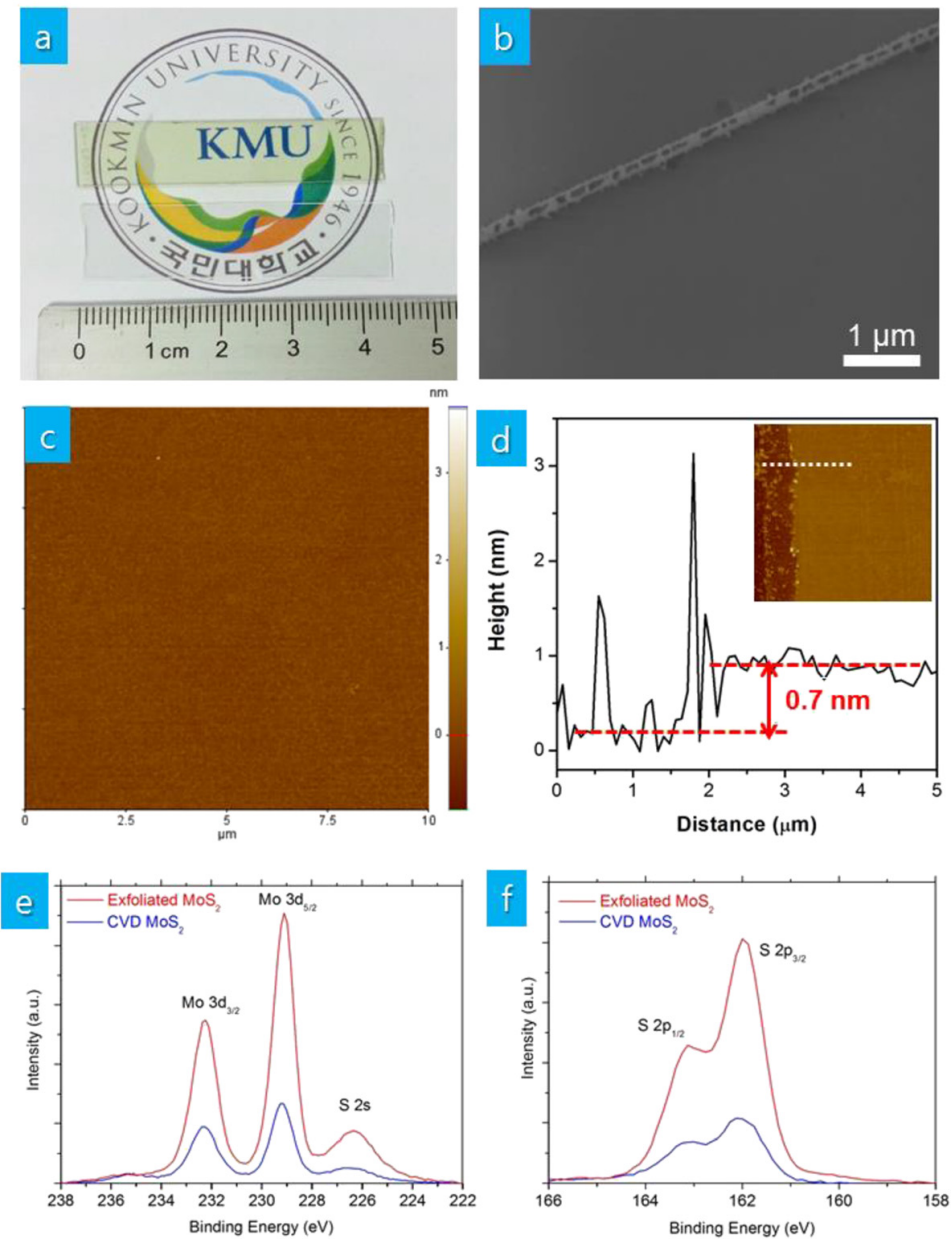
**a** As-grown MoS_2_ thin films on a sapphire substrate in comparison with a bare sapphire substrate, **b** SEM image of an intentionally scratched MoS_2_ thin films, **c** AFM image, **d** cross-sectional AFM image along the dotted line in the AFM image, **e**, **f** XPS spectra of MoS_2_ thin films along with those of mechanically exfoliated MoS_2_ flakes

MoS_2_ thin films deposited on sapphire substrates are light yellow-green in color exhibiting obvious color contrast with transparent bare sapphire substrates as shown in Fig. 1a. The continuous formation of MoS_2_ thin films is observed up to 4 cm. The existence of MoS_2_ thin films on substrates can be confirmed by the color contrast in the SEM image of an intentionally scratched sample in Fig. 1b. When MoO_3_ and S powders are used as precursors, triangular-shaped discontinuous clusters of either single-layer MoS_2_ or mixtures of single- and few-layer MoS_2_ are typically observed in literature [12]. Similarly, triangular-shaped clusters or regions of bilayer MoS_2_ can be observed in this investigation when process conditions are not optimized (see Additional file 1). However, with optimized process conditions, such triangular clusters of MoS_2_ cannot be found in our MoS_2_ thin films as shown Fig. 1b. The absence of triangular clusters of MoS_2_ is further confirmed by AFM image in Fig. 1c. The AFM measurement on the scratched sample in Fig. 1d shows thickness of 0.7 nm corresponding to that of single-layer MoS_2_. As MoS_2_ growth is known to be sensitive to the localized concentration of precursors [20], the combination of the low pressure, distance between precursors and substrates, and temperature used in this investigation may result in uniform nucleation and growth of MoS_2_. Figure 1e, f compares XPS of the deposited MoS_2_ thin films with that of bulk MoS_2_ single crystals. The existence of Mo^4+^ (Mo 3*d*_5/2_ orbital at 229.1 eV and Mo 3*d*_3/2_ orbital at 232.3 eV) and S^2−^ (S 2*p*_3/2_ orbital at 162.0 eV and S 2*p*_1/2_ orbital at 163.1 eV) is clearly seen in our thin films.

**Fig. 2:**
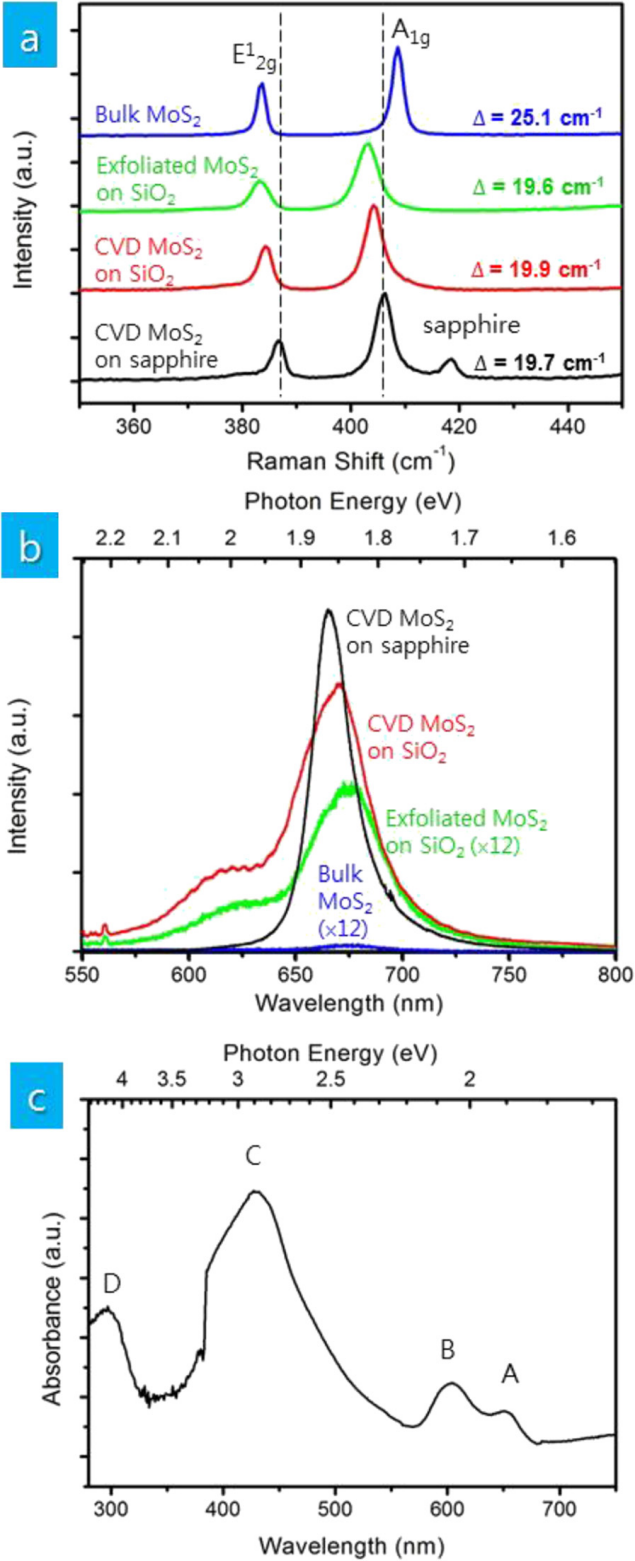
**a** Raman and **b** PL spectra of MoS_2_ thin films on sapphire and SiO_2_/Si, mechanically exfoliated MoS_2_ flakes on SiO_2_/Si, and bulk MoS_2_ crystals and **c** UV-visible spectrum of MoS_2_ thin films on sapphire

To confirm the thickness of deposited MoS_2_ thin films, we measure the Raman spectra of deposited MoS_2_ thin films. Figure 2a shows the two characteristic Raman A_1g_ and E^1^_2g_ modes of four different MoS_2_ samples—CVD MoS_2_ thin films on sapphire substrates, CVD MoS_2_ thin films transferred on SiO_2_/Si substrates, mechanically exfoliated single-layer MoS_2_ flakes on SiO_2_/Si substrates, and bulk MoS_2_ single crystals. The frequency difference between A_1g_ and E^1^_2g_ modes (Δ) of MoS_2_ is related to its thickness [21]. Except bulk single crystals (Δ = 25.1 cm^−1^), all other MoS_2_ samples possess Δ between 19.6 and 19.9 cm^−1^ suggesting single-layer MoS_2_. It needs to be mentioned that the positions of A_1g_ and E^1^_2g_ modes are slightly shifted for single-layer MoS_2_ on SiO_2_/Si substrates (both transferred CVD films and mechanically exfoliated MoS_2_ flakes). This is due to the effect of underlying SiO_2_/Si substrates as substrates can strongly affect the Raman and PL emission of single-layer MoS_2_ [22].

The thickness of MoS_2_ thin films is further confirmed by PL spectra. While the indirect bandgap of multilayer MoS_2_ does not allow PL emission, the direct bandgap of single-layer MoS_2_ allows PL emission [23, 24]. Figure 2b shows the PL spectra of the same four MoS_2_ samples—CVD MoS_2_ thin films on sapphire substrates, CVD MoS_2_ thin films transferred on SiO_2_/Si substrates, mechanically exfoliated single-layer MoS_2_ flakes on SiO_2_/Si substrates, and bulk MoS_2_ single crystals. While the emission intensity completely disappears for bulk MoS_2_, our single-layer MoS_2_ thin films on sapphire show a PL emission peak at 1.88 eV confirming they are single-layer MoS_2_. The PL spectrum of our single-layer MoS_2_ thin films on sapphire is different from that of mechanically exfoliated single-layer MoS_2_ flakes on SiO_2_/Si. Two emission peaks are observed for mechanically exfoliated single-layer MoS_2_ flakes on SiO_2_/Si at 1.85 and 2.00 eV known as A and B direct excitonic transitions [23, 24]. The shift of emission peak A and the absence of emission peak B in our single-layer MoS_2_ thin films on sapphire are due to the effect of the underlying substrate [22]. The consistent PL emission spectrum from transferred single-layer MoS_2_ on SiO_2_/Si with that of mechanically exfoliated single-layer MoS_2_ flakes on SiO_2_/Si supports this.

The UV-visible absorption spectrum in Fig. 2c shows A and B absorption due to excitonic transitions along with C and D absorption associated with van Hove singularity [25, 26]. As the existence of van Hove singularity can enhance light-matter interactions, single-layer MoS_2_ thin films may be suitable for photovoltaic cells and photodetectors due to enhanced photon absorption and electron-hole creation.

**Fig. 3:**
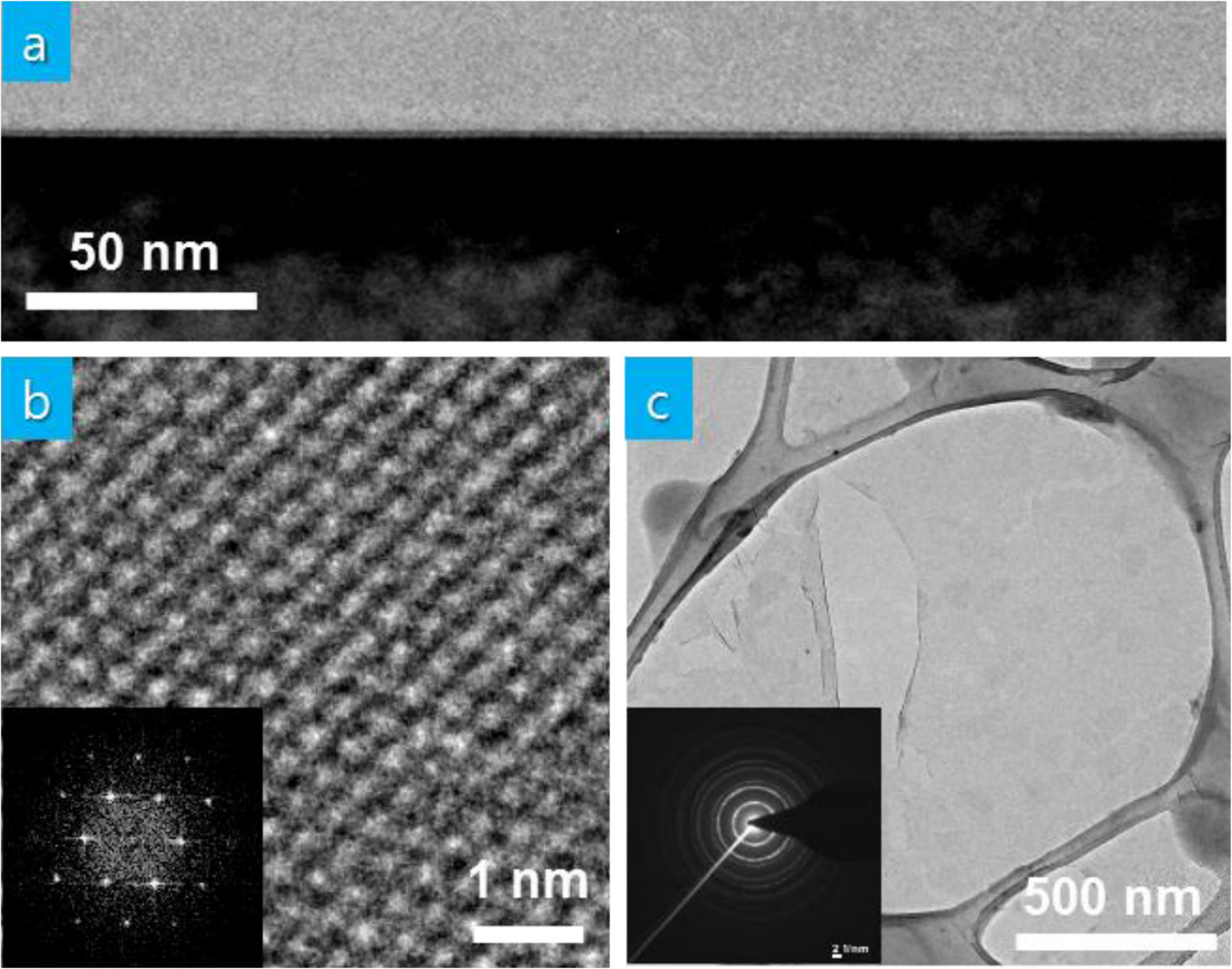
**a** Cross-sectional TEM image of MoS_2_ thin films on sapphire, **b** high-magnification plan-view TEM image along with fast Fourier transformation pattern, **c** low-magnification plan-view TEM image along with SAED pattern MoS_2_ thin films

We also perform TEM analysis to obtain information on the crystallinity of the single-layer MoS_2_ thin films. The low-magnification bright-field TEM cross-sectional image in Fig. 3a shows continuous single-layer MoS_2_ thin films on sapphire substrates. The high-resolution plan-view image in Fig. 3b and the corresponding fast Fourier transformation pattern in its inset reveal the hexagonal crystal structure of single-layer MoS_2_ thin films. The estimated interplanar spacing of (100) and (110) planes is ~0.28 and ~0.16 nm, respectively, which is in good agreement with literature [12]. The selected area electron diffraction (SAED) pattern obtained from the low-magnification plan-view image in Fig. 3c shows multiple rings confirming the polycrystalline nature of the MoS_2_ thin films.

**Fig. 4:**
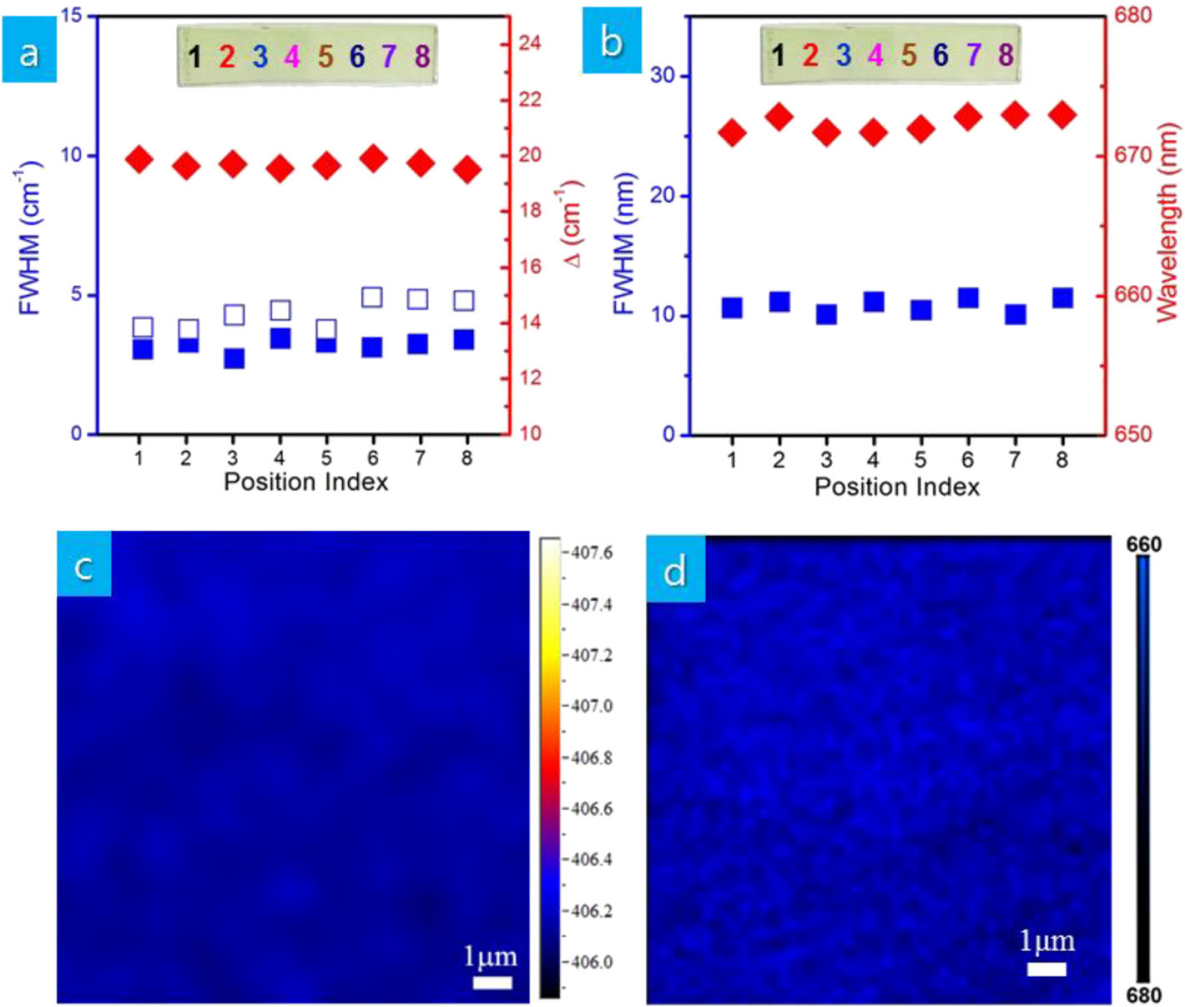
**a** FWHM of Raman E^1^_2g_ and A_1g_ modes along with Δ (red diamond: Δ, white square: FWHM of Raman A_1g_ mode, blue square: FWHM of Raman E^1^_2g_ mode) and **b** FWHM along with wavelength of PL emission (red diamond: wavelength of PL emission, blue square: FWHM of PL emission) measured at eight different positions of MoS_2_ thin films shown in the inset, **c** mapping of Raman A_1g_ mode frequency, and **d** mapping of PL emission wavelength of MoS_2_ thin films

For more quantitative assessment of the thickness uniformity, Raman and PL spectra are measured at eight different positions of a 4-cm-wide single-layer MoS_2_ thin film (insets of Fig. 4a, b). Figure 4a, b shows negligible variation of the measured full-width at half maximum (FWHM) of Raman A_1g_ and E^1^_2g_ modes, Δ, FWHM of PL emission peak, and PL emission wavelength. Figure 4c, d shows the measured Raman A_1g_ mode frequency and PL emission wavelength based on mapping over an area of 10 μm × 10 μm, respectively. Measured Raman A_1g_ mode frequency and PL emission wavelength exist in a range of 406.1–406.3 cm^−1^ and 659–662 nm, respectively. These results suggest that the thickness of our single-layer MoS_2_ thin films is uniform across the substrate.

## Conclusions

In summary, we synthesized uniform large-area thin films of single-layer MoS_2_ on sapphire substrates by CVD based on MoO_3_ and S precursors. The as-grown thin films were composed of single-layer MoS_2_ and continuous for more than 4 cm without triangular-shaped clusters of MoS_2_. The chemical configuration, thickness, thickness uniformity, and crystalline quality of MoS_2_ thin films were confirmed by XPS, AFM, Raman and PL spectra, and TEM analysis. The optical absorbance measurement further suggested the existence of van Hove singularity at high energy. These results will help scale up the growth of two-dimensional TMDs, providing potentially important implications on realizing the promising potential of high-performance MoS_2_ devices such as thin-film transistors, sensors, and photodetectors.

## Additional file

Additional file 1:
**Formation of nonuniform MoS**_**2**_ **clusters.** (DOCX 379 kb)
