# Supplementary material for: Large-Area Growth of Uniform Single-Layer MoS2 Thin Films by Chemical Vapor Deposition
Source: Nanoscale Res Lett. 2015 Oct 6;10:388. doi: 10.1186/s11671-015-1094-x (PMC4595407; doi:10.1186/s11671-015-1094-x)
Supplement: Additional file 1: — Formation of nonuniform MoS 2 clusters. (DOCX 379 kb) [file 11671_2015_1094_MOESM1_ESM.docx]

**Additional file 1**

Large-area growth of uniform single-layer MoS_2_ thin films by chemical vapor deposition

*By* Seung Hyun Baek, Yura Choi, and Woong Choi^*^

**1. Formation of triangular-shaped single-layer MoS_2_ clusters**

While it needs more studies to fully understand the nucleation and growth process of MoS_2_ thin films, our MoS_2_ films grow from triangular clusters. When growth conditions are not optimized, the triangular-shaped clusters of single-layer MoS_2_ can be obtained instead of continuous thin films. A representative SEM image is shown in Figure S1. The thickness of a triangular-shaped cluster (~0.7 nm) measured by AFM confirms single-layer MoS_2_.


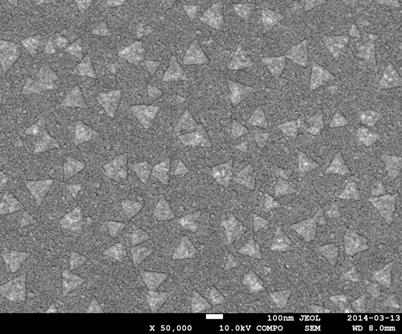

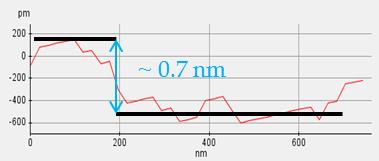


b

a

Figure S1. (a) Triangular-shaped clusters of single-layer MoS_2_ observed by SEM; (b) The thickness of a triangular-shaped cluster measured by AFM confirms single-layer MoS_2_.

**2. Formation of bilayer MoS_2_ clusters**

Regions of bilayer MoS_2_ can be obtained in the middle of continuous single-layer MoS_2_ thin films when the growth conditions are not optimized. Figure S2 shows an SEM image of an intentionally-scratched sample. The frequency difference between E^1^_2g_ and A_1g_ Raman modes indicates bilayer MoS_2_ (darker region) within continuous thin films of single-layer MoS_2_ (brighter region). Although more studies are needed to pinpoint exactly what makes our MoS_2_ thin films uniform single-layer, the localized concentration of precursors seems to be most influential in our investigation. For instance, even a minor change in the distance between precursors and substrates could strongly influence the uniformity of MoS_2_ thickness.


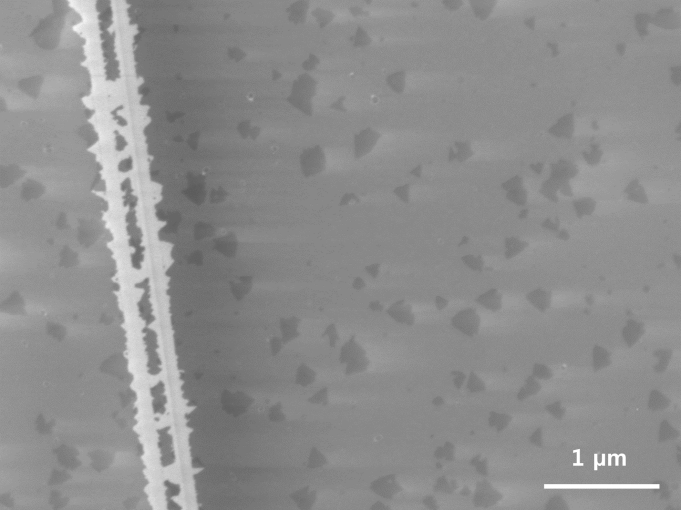


b

a

Figure S2. (a) SEM image showing an intentionally-scratched MoS_2_ thin film exhibiting darker regions within continuous thin films (brighter region); (b) The frequency difference between E^1^_2g_ and A_1g_ Raman modes indicates that the darker regions in (a) are bilayer MoS_2_ and brighter region in (a) is single-layer MoS_2_.
